# Granzymes are necessary for suppressive function of regulatory T cells

**DOI:** 10.1186/1546-0096-10-S1-A108

**Published:** 2012-07-13

**Authors:** Dominic O Co, Dipica Haribhai, Erica G Schmitt, Jennifer Ziegelbauer, William J Grossman, James Verbsky, Calvin Williams

**Affiliations:** 1 Baxter Healthcare Corporation, Deerfield, IL, USA; 2 Medical College of Wisconsin, Milwaukee, WI, USA

## Purpose

Regulatory T (Treg) cells are an essential subset of CD4+ T cells that induce and maintain immunological tolerance. Preclinical animal models have demonstrated that adoptive transfer of Treg cells can prevent or cure diabetes, multiple sclerosis (EAE), inflammatory bowel disease, lupus, arthritis, and graft versus host disease. Defects in Treg cell function and number have been described in a number of different human autoimmune diseases including diabetes, multiple sclerosis, rheumatoid arthritis and juvenile idiopathic arthritis. These data suggest that manipulation of Treg cells may be a useful therapeutic intervention. Treg cells are marked by expression of the forkhead/winged helix transcription factor Foxp3, which is essential for their regulatory functions. Treg cells are well known to mediate their immunomodulatory effects through TGF-β, IL-10 and CTLA-4. Recent work has also suggested a role for the granule/exocytosis pathway in Treg cell suppressive function. Granzyme A and granzyme B (gzm A and gzm B, respectively) mRNAs are expressed at high levels in Treg cells, and Treg cells from gzm B–/– mice showed defects in suppression in vitro. To further examine the role of granzymes in the function of Treg cells in vivo, we used a T cell transfer model of colitis. In this model, naive CD45RBhigh CD4+ T cells transferred to RAG–/– mice and undergo homeostatic expansion and activation.This process results in colitis manifested clinically by weight loss and shortened survival. Previous work has shown that a subset of the transferred conventional T cells develop into Foxp3+ iTreg cells and that these iTreg cells are necessary to mitigate colitis when present together with natural Treg cells (nTreg) cells derived in the thymus.

## Methods

CD45RBhigh T cells from mice expressing a Foxp3-EGFP fusion protein and additionally deficient in gzm A or gzm B are transferred to RAG-/- mice.In these mice, EGFP expression marks iTreg which have developed in situ. Mice were weighed twice weekly and euthanized when moribund or when they have lost more than 15-20% of their initial weight. Mesenteric lymph nodes T cells were analyzed for expression of EGFP.

## Results

Mice receiving gzm A or B deficient cells lose weight faster and die earlier than mice transferred with WT cells. Mice transferred with CD45RBhigh CD4+ T cells from granzyme-deficient mice also appear to have lower numbers of iTreg cells.

## Conclusion

These results suggest that granzymes are important in the function of iTreg cells and implicate granzymes in iTreg cell survival. In current experiments we are using granzyme-deficient nTreg and iTreg cells for adoptive transfer immunotherapy of colitic mice to more specifically dissect the role of granzymes in Treg cell function.

## Disclosure

Dominic O. Co: None; Dipica Haribhai: None; Erica G. Schmitt: None; Jennifer Ziegelbauer: None; William J. Grossman: None; James Verbsky: None; Calvin Williams: None.

